# Synthetic Heparan Sulfate Mimetic Polymer Enhances Corneal Nerve Regeneration and Wound Healing after Experimental Laser Ablation Injury in Mice

**DOI:** 10.3390/polym14224921

**Published:** 2022-11-15

**Authors:** Ignacio Alcalde, Cristina Sánchez-Fernández, Susana Del Olmo-Aguado, Carla Martín, Céline Olmiere, Enol Artime, Luis M. Quirós, Jesús Merayo-Lloves

**Affiliations:** 1 Instituto Universitario Fernández-Vega, Fundación de Investigación Oftalmológica, University of Oviedo, 33012 Oviedo, Spain; 2 Instituto de Investigación Sanitaria del Principado de Asturias (ISPA), 33011 Oviedo, Spain; 3 Department of Functional Biology, University of Oviedo, 33006 Oviedo, Spain; 4 Laboratoires Thea S.A.S., 63000 Clermont-Ferrand, France

**Keywords:** wound healing, nerve regeneration, cornea, transparency, extracellular matrix, heparan sulfate mimetic polymer

## Abstract

(1) Background: Abnormal corneal wound healing compromises visual acuity and can lead to neuropathic pain. Conventional treatments usually fail to restore the injured corneal tissue. In this study, we evaluated the effectiveness of a synthetic heparan sulfate mimetic polymer (HSmP) in a mouse model of corneal wound healing. (2) Methods: A surgical laser ablation affecting the central cornea and subbasal nerve plexus of mice was used as a model of the wound-healing assay. Topical treatment with HSmP was contrasted to its vehicle and a negative control (BSS). Corneal repair was studied using immunofluorescence to cell proliferation (Ki67), apoptosis (TUNEL assay), myofibroblast transformation (αSMA), assembly of epithelial cells (E-cadherin) and nerve regeneration (β-tubulin III). (3) Results: At the end of the treatment, normal epithelial cytoarchitecture and corneal thickness were achieved in HSmP-treated animals. HSmP treatment reduced myofibroblast occurrence compared to eyes irrigated with vehicle (*p* < 0.01) or BSS (*p* < 0.001). The HSmP group showed 50% more intraepithelial nerves than the BSS or vehicle groups. Only HSmP-treated corneas improved the visual quality to near transparent. (4) Conclusions: These results suggest that HSmP facilitates the regeneration of the corneal epithelium and innervation, as well as restoring transparency and reducing myofibroblast scarring after laser experimental injury.

## 1. Introduction

Corneal injuries may occur due to viral infection, chemical burns, surgical procedures, corneal dystrophy, diabetes or trigeminal nerve damage and remain difficult to treat [1]. Abnormal wound healing in patients may involve the chronicity of corneal lesions, which can result in permanent corneal opacification, irregular astigmatism due to corneal scarring, intense neuropathic pain or even corneal perforation [2]. Corneal opacification is one of the main causes of blindness worldwide after cataracts [3].

The mechanisms regulating the normal physiological renewal of the corneal tissue involve proliferation, migration and differentiation of cells [4]. In addition, sensory nerves play a crucial role in maintaining the cornea in a healthy state, as well as promoting wound healing [5,6,7]. After corneal injury, epithelial cells proliferate and migrate to cover the wound bed before differentiating into a new multilayered epithelium [4]. Keratocytes beneath the damaged area shift into proliferating activated cells (myofibroblasts), develop actin contractile elements [8,9,10] and migrate to the site of injury [11,12]. They are responsible for wound contraction and extracellular matrix (ECM) deposition [13]. Injury of trigeminal nerve fibers innervating the cornea causes neuropathic keratitis, which can result in permanent lesion of the cornea, perforation and/or stromal melting [7,14]. After surgical or chemical sensory denervation of the cornea, the process of corneal wound healing is delayed [14,15,16]. Thus, regeneration of injured sensory axons is necessary to recover the full functionality of the cornea [17]. In an injured cornea, ECM produced by myofibroblasts reduces keratan sulfate proteoglycans (KSPG), which results in reduced transparency and stromal scarring [18]. It is well-known that heparan sulfate proteoglycans, such as syndecans, promote axon regeneration by stabilizing growth cone migration [19], while chondroitin sulfate proteoglycans inhibit nerve regeneration [20].

The biological process of corneal wound healing, the synthesis of new epithelium and stroma and sensory nerve regeneration are mediated by growth factors, including epithelial growth factor (EGF), keratinocyte growth factor, hepatocyte growth factor, fibroblast growth factor (FGF), transforming growth factor-β (TGF-β) and platelet-derived growth factors (PDGFs), among others [21,22,23]. In addition, proteoglycans in the ECM and on the cell surface are key players for the stabilization of growth factors [24] and cell communication [25], regulating cell migration and axonal regeneration guidance [19,25]. 

Artificial tears are the most widely used treatment for the management of ocular surface damage, with good outcomes regarding lubrication and relief of desiccation symptoms [2,3]. However, they lack the biological components of natural tears and often contain preservatives and other additives that may potentially induce toxic or allergic reactions and worsen dry eye symptoms [26,27]. Medical treatments usually involve the inhibition of inflammation and modulation of the immune response and present important side effects [28]. Alternative topical methods, including autologous serum [29] and autologous plasma rich in growth factors (PRGF) [30,31], have been shown to enhance ocular surface wound healing. PRGF has been reported to improve wound healing and corneal transparency in an experimental animal model [30,31]. 

**Scheme 1:**
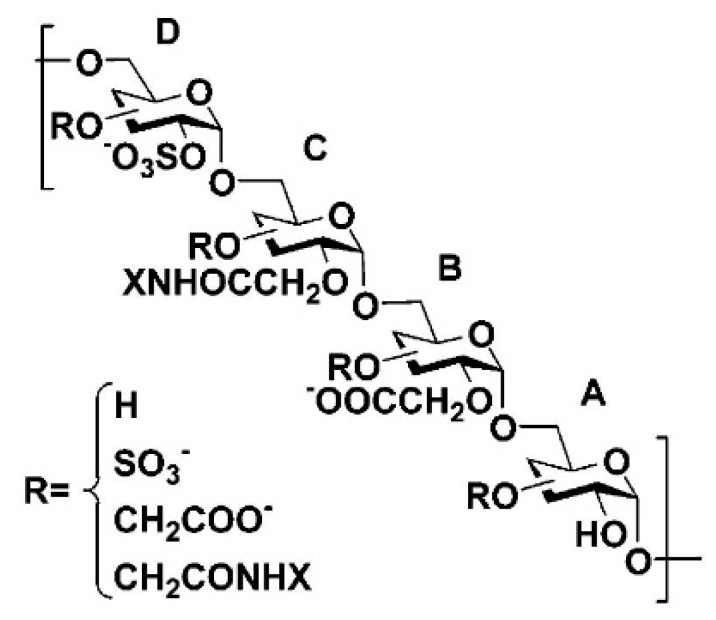
General structure of heparan sulfate mimetic polymers. Four differently substituted units (A, B, C and D) can be present in HSm polymers (adapted from [32]).

New types of matrix therapy agents have provided encouraging results, accelerating, for example, the healing of chronic skin ulcers of diabetic or vascular origin [1]. Large polymers have been designed to mimic glycosaminoglycans and are used as regenerating agents [32]. Regenerating agents (RGTAs) are heparan sulfate mimetic polymers (HSmPs) that replace destroyed heparan sulfate molecules, creating a cellular microenvironment favorable to healing [1]. RGTAs are engineered high-sulfated biopolymers (Scheme 1) that mimic heparan sulfates bound to ECM proteins [33], avoiding their proteolysis and acting as a protector and stabilizer of the actions of heparin-binding growth factors [34,35,36,37], and they are known to stimulate wound healing under various conditions in different in vivo systems [38,39,40,41]. Manipulation of the extracellular environment by adding high sulfated synthetic glycosaminoglycan mimetic can improve the regenerative capacity of corneal cells.

In this study, we evaluated the regenerative effects of RGTA drops on the proliferation, scarring and nerve regeneration during corneal wound healing in mice cornea after a lesion induced by laser ablation using an excimer laser.

## 2. Materials and Methods

### 2.1. Animals

A total of 105 mice (C57BL/6) aged 3 months and purchased from Charles River Laboratory (L’Arbresle Cedex, France) were used. Animals were handled and housed according to the ARVO Statement for the Use of Animals in Ophthalmic and Vision Research, the applicable guidelines of the EU (2003/65/EC) and the Spanish Government (RD 53/2013) and the Ethics Committee of the University of Oviedo. On the day of sacrifice, animals were euthanized by sodium pentobarbital overdose (Dolethal, Vétoquinol, Lure, France) injected intraperitoneally, under general anesthesia. Immediately thereafter, eyes were enucleated and processed for immunofluorescence studies.

Mice were divided into three groups depending on the treatment: (1) HSmP mice treated with regenerating agent (RGTA, Cacicol^®^, Théa Laboratoires, Clermont-Ferrand, France; n = 35); (2) Dx mice treated with the vehicle of HSmP (Dextran T40 in physiological saline solution; n = 35) and (3) BSS mice treated with balanced salt solution (BSS, Sterile Irrigating Solution, Alcon Laboratories, Inc., Fort Worth, TX, USA; n = 35). Animals treated only with BSS were considered as a negative control. BSS is commonly used as irrigating solution in clinic and as a control in experimental designs due to its biological inactivity [42,43,44]. Only the right eye of each mouse was surgically injured and the contralateral eye served as uninjured control. Mice in the BSS, vehicle (Dx) and HSmP groups received the treatment topically in both eyes twice: one drop (5 µL) 1 h after the induction of the surgical injury and one drop 48 h later. 

### 2.2. Induction of Corneal Injury by Laser Ablation

Corneal injury was performed as previously described [17,30,31,45,46,47]. Right eyes were subjected to a photorefractive keratectomy (PRK) laser ablation surgical procedure with a 2.0 mm ablation zone on the central cornea and a depth of 45 µm (including the epithelium) with a VISX Star S3 excimer laser (Johnson & Johnson, Santa Ana, CA, USA) used only for animal research. Before surgical procedures, mice were deeply anaesthetized by intraperitoneal injection of a mixture of ketamine hydrochloride (80 mg/Kg; Imalgene 1000, Merial Laboratorios S.A., León, Spain) and xylazine hydrochloride (5 mg/Kg; Rompun, Bayer Hispania S.L., Barcelona, Spain), followed by topical application of 0.5% tetracaine clorhydrate and 1 mg of oxybuprocaine (Colircusí Anestésico Doble, Alcon S.A., Barcelona, Spain).

### 2.3. Clinical Course

Corneal wound healing was examined under a Leica S6D stereoscopic microscope (Leica Microsystems, Wetzlar, Germany) immediately after surgery and at 1, 2, 3, 7 and 15 days after PRK surgery (0 H, 1 D, 2 D, 3 D, 7 D and 15 D, respectively). The fluorescein-staining test (Alcon S.A., Barcelona, Spain) was used to visualize the corneal epithelial defect. FIJI image analysis software (ImageJ 1.52d; National Institutes of Health, Bethesda, MD, USA) was used to calculate the area (mm^2^) of the epithelial defect. The level of opacity (haze) in the cornea was assessed by two researchers observing under a stereomicroscope according to OECD Test Guidance 405 Annex I scale (score 0 (clear) to 4 (severely dense opacity)) [48].

### 2.4. Tissue Processing and Microscopy

Five eye globes from each group and time point were fixed by immersion in buffered 4% paraformaldehyde (4% PF) for 1 h at room temperature, cryoprotected in 30% sucrose, embedded in OCT compound (Optimum Cutting Temperature; Tissue-Teck, Sakura, Tokyo, Japan) and snap frozen in liquid nitrogen. Transversal sections (5 µm) were obtained with a Microm HM550 cryostat (Microm International GmbH, Walldorf, Germany) through the central region of the cornea for immunofluorescence analysis using specific antibodies to study proliferation (rabbit polyclonal antibody to Ki67; 1:500; Abcam, Cambridge, UK), myofibroblast transformation (rabbit polyclonal antibody to αSMA 1:200; Abcam), assembly of epithelial cells (rabbit polyclonal antibody to E-cadherin 1:200; Santa Cruz Biotechnology, Inc., Santa Cruz, CA, USA) and nerve regeneration (rabbit monoclonal antibody to neuronal class III β-tubulin (β-tubulin III) 1:500; Abcam). Immunofluorescence assays were performed as described previously [17,30,45]. Samples were incubated overnight with the corresponding antibody and revealed with complementary Alexa Fluor 594 anti-rabbit secondary antibody (1:500; Molecular Probes, Eugene, OR, USA). Nuclei were counterstained with DAPI (4′,6-diamidino-2-phenylindole; 2 µg/mL; Molecular Probes). A TUNEL histochemical assay (dUTP Nick End Labeling; Promega BioSciences LLC, San Luis Obispo, CA, USA) was performed according to the manufacturer’s instructions to visualize cells undergoing apoptosis. Sections were examined under a Leica DM 6000 fluorescence microscope equipped with a digital image capture system (LASX, Leica Microsystems GmbH, Wetzlar, Germany).

### 2.5. Cell Counting

Three equidistant sections (at 50 µm intervals) obtained from the central region of five corneas for each experimental group were used to quantify Ki67^+^ cells and TUNEL-labeled cells in the entire corneal section (in both the epithelium and the stroma) with the Cell Counter utility of FIJI software. Corneal αSMA^+^ myofibroblasts were counted by two independent observers. Every positive cell with clearly identifiable nuclei (DAPI stained in blue) was counted on five nonoverlapping corneal regions of 224.14 × 167.38 μm in size. Each microscope field comprised a column of central corneal tissue extending from the anterior epithelium to the posterior stromal surface. The density of regenerating nerve fiber terminals (β-tubulin III^+^) in the epithelium was calculated as the number of terminals/linear mm based on the “epidermal nerve fiber density” index. Measures of the thickness of the cornea were made on the same sections used for immunofluorescence analysis using the FIJI software utility “straight line” and automatic measurement. All morphometric analysis was carried out using FIJI software.

### 2.6. Whole-Mounted Cornea Immunofluorescence

Five eyeballs from each group of mice were freshly isolated at 7 D, dissected and fixed 1 h at room temperature (RT) in 4% PF and washed in 0.1 M PBS, pH 7.4 (PBS). They were blocked for 1 h with 5% BSA, 5% goat serum, 0.2% sodium azide and 0.3% Triton X-100 in PBS (PBS-Triton; all products from Merck KGaA, Darmstadt, Germany). Corneas were incubated for 24 h at RT in the presence of rabbit anti–neuronal class III β-tubulin (1:500; Abcam, Cambridge, UK). The samples were then incubated for 24 h at RT in the presence of anti-rabbit IgG Alexa Fluor 594 secondary antibody (1:500; Molecular Probes). Afterwards, samples were rinsed three times with washing solution followed by incubation for 10 min at RT with DAPI, and, finally, they were mounted with fluorescent mounting medium (DAKO, Glostrup, Denmark).

### 2.7. Sholl Analysis

For Sholl analysis, images were acquired using the Leica TCS-SP2-AOBS spectral confocal microscope (Leica Microsystems). Leica LAS X software was used to fuse the adjacent tiles and produce maximum intensity projections. The adjacent image tiles were captured with overlap to ensure proper tiling. All images were acquired using the same intensity settings. ImageJ Sholl analysis v3.2.7 plugin from FIJI [49] was used to calculate the number of regenerative sensory axons in the injured region. Intersecting fibers were automatically counted at 100, 300, 500, 700, 900 and 1100 µm from the corneal center.

### 2.8. Statistical Analysis

All experiments were conducted in a masked fashion. Data extracted from the comparison analysis were expressed as means ± standard error of the mean (SEM). The differences among groups for each parameter were assessed by analysis of variance (ANOVA) followed by the Student–Newman–Keuls test. A *p* value of <0.05 was considered statistically significant. Prism 6 statistical software (GraphPad Software Inc., San Diego, CA, USA) was used to run the analysis.

## 3. Results

### 3.1. Wound Healing

**Figure 1:**
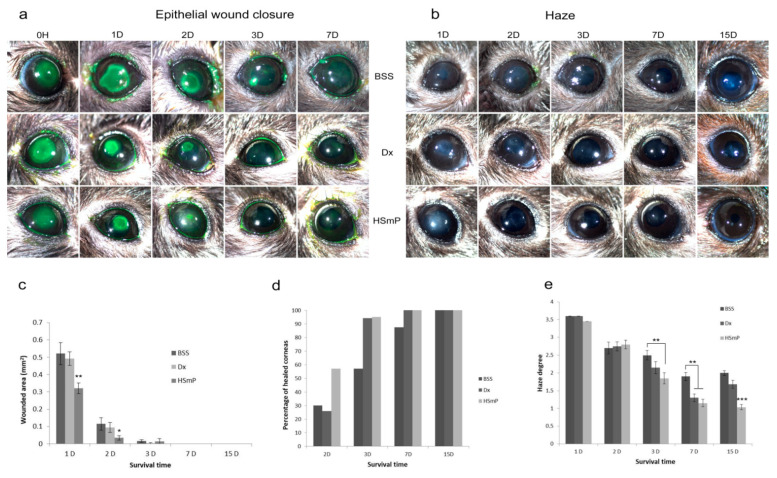
Macroscopic findings for the injured ocular surface. (**a**) The fluorescein-staining test demonstrated the epithelial defect in a temporal sequence. No fluorescein staining was recorded at 15 D. (**b**) The transparency of the cornea was reduced in the first two postsurgical days. The HSmP group showed transparent corneas at 15 D (magnification in (**a**,**b**): 12.5×). (**c**) Wound healing was accelerated after application of one drop of HSmP. (**d**) Zero percent of the corneas in the HSmP group had healed completely at 48 h. Some BSS treated eyes presented incomplete wound closure at day 7. (**e**) The grade of transparency was significantly improved in HSmP-treated animals 15 days after injury. Dx- and BSS-treated corneas showed no transparency recovery by the end of the experiment (* = *p* < 0.05; ** = *p* < 0.01; *** = *p* < 0.001).

Laser induced wounds were strongly stained with fluorescein from 0 H, showing averaged wounded areas of 2.76 ± 0.28 mm^2^. The speed of wounded area reduction was significantly improved with HSmP treatment compared to Dx and BSS (*p* < 0.01) at day 1 and day 2 (*p* < 0.05; Figure 1a,c)). In addition to wound healing area closure, HSmP-treated epithelia were preferentially found to be completely healed at day 2. A total of 60% of eyes showed no fluorescein staining in the HSmP group compared to only 26% in the Dx group and 30% in the BSS group. At day 7, all eyes from HSmP and Dx groups were totally healed, while 12.5% of the animals in the BSS group still showed epithelial lesions. At the end of the experiment (day 15), all eyes showed no fluorescein staining (Figure 1d).

### 3.2. Corneal Transparency

Upon laser ablation, injured eyes of all groups (HSmP, Dx and BSS) showed high levels of corneal opacity (day 1). This opacity made it impossible to observe structures of the anterior chamber and the iris in all groups (Figure 1b), showing an intense degree of haze (about 3.5 in the OECD TG405 scale). After 72 h, HSmP-treated corneas showed a significant reduction in the opacity compared to the BSS group (*p* < 0.01), scoring <2.0. The Dx group showed a grade of opacity over 2.3 and did not differ significantly from BSS (Figure 1e). The degree of transparency at day 7 was also greater in corneas treated with HSmP compared to BSS (*p* < 0.001), with HSmP-treated mice showing only a mild degree of haze. At the end of the experiment (day 15), haze degree in BSS corneas were not reduced compared to day 7, and animals in the Dx group reversed the course of healing and showed a higher degree of opacity. At this time-point, HSmP-treated animals showed near transparent corneas, significantly more transparent than Dx or BSS corneas (*p* < 0.001).

### 3.3. Histopathological Analysis

To further understand the macroscopic features of wound healing upon HSmP treatment, we performed a series of histopathological and morphometric analysis. Measures of the corneal thickness at the center of the cornea showed that eyes treated with HSmP at day 7 presented values statistically equivalent to those measured in uninjured corneas (98.94 ± 1.62 µm in HSmP group vs. 105.47 ± 1.60 µm in untreated healthy corneas; n.s.). Compared to BSS and Dx groups, corneal thickness was significantly greater in the HSmP group (*p* < 0.001) (Table 1).

#### 3.3.1. Cell Proliferation

**Figure 2:**
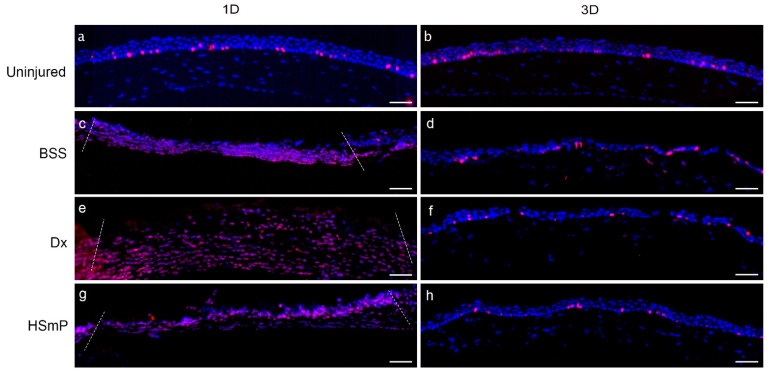
Proliferative cells at day 1 and day 3 after injury. In red, cells expressing Ki67 as a marker of proliferation. Uninjured corneas (**a**,**b**) showed a low number of proliferative cells restricted to the basal layer of the epithelium. HSmP-treated corneas (**g**) showed a lower number of proliferative cells compared to vehicle (**e**) and BSS (**c**) one day after injury. Numbers of Ki67+ cells were greatly reduced at 72 h in all groups (**d**,**f**,**h**). HSmP presented a lower number of Ki67+ cells. Dashed lines mark the border of the initial injury in the 1 D series of images (scale bars: 50 µm).

During the first 72 h, Ki67^+^ dividing cells were numerous in the stroma, resulting in a high cellular density in the injured area. Apart from fibroblasts, many Ki67^+^ inflammatory cells could be identified by the characteristic shape of their nuclei (neutrophils and lymphocytes). There were also proliferative cells in the epithelium at the periphery of the wound (Figure 2).

Corneas treated with HSmP showed a significantly lower density of Ki67^+^ cells (*p* < 0.05) 24 h after injury compared to Dx or BSS (Figure 2 and Table 2). The density of Ki67-positive cells decreased rapidly between 24 and 48 h in all groups of injured mice (Table 2). The lowest density of cells on day 2 was found in HSmP-treated corneas (Table 2), with a marked difference from BSS-treated corneas (*p* < 0.001). By day 3, HSmP eyes showed a low density of dividing cells compared to Dx (*p* < 0.001) and BSS (*p* < 0.01). Dividing cells were restricted almost exclusively to the epithelium in HSmP group while Ki67^+^ cells were still present in the stroma in Dx and BSS groups (Figure 2d–f). Values of the dividing cell count were close to normal by day 7 in all groups, with no statistical differences between them (Table 2).

#### 3.3.2. Apoptosis

**Figure 3:**
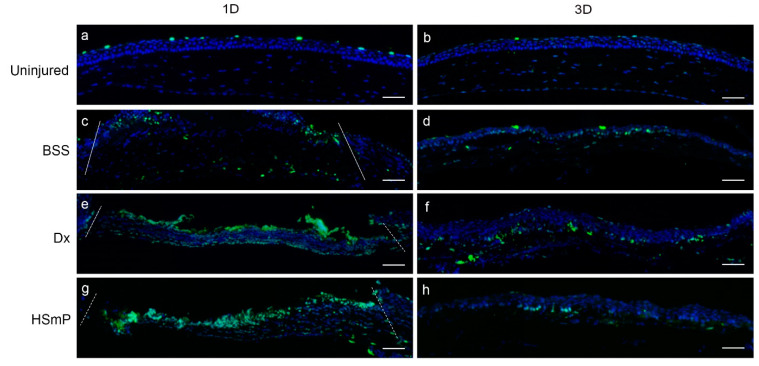
TUNEL-positive profiles (in green) in the laser-ablated region (1 day and 3 days after surgery). Uninjured corneas (**a**,**b**) showed a low number of apoptotic cells restricted to the outermost layer of the epithelium. One day after injury, the BSS-treated group (**c**) showed a higher number of apoptotic cells compared to Dx (**e**) or HSmP (**g**). Number of TUNEL+ cells was greatly reduced at 72 h in all groups (**d**,**f**,**h**). Dashed lines mark the border of the initial injury in the 1 D series of images (scale bars: 50 µm).

The maximum rate of apoptotic events was found within the first 48 h after the induction of the wound in all groups. The maximum density of TUNEL^+^ cells at day 1 was in the BSS group (Figure 3 and Table 2) while the HSmP-treated group had the lowest density of apoptotic cells (*p* < 0.01). TUNEL positive cells were observed both in the epithelium surrounding the wound and in the central corneal stroma, where epithelium was absent at day 1 after injury (Figure 3).

**Figure 4:**
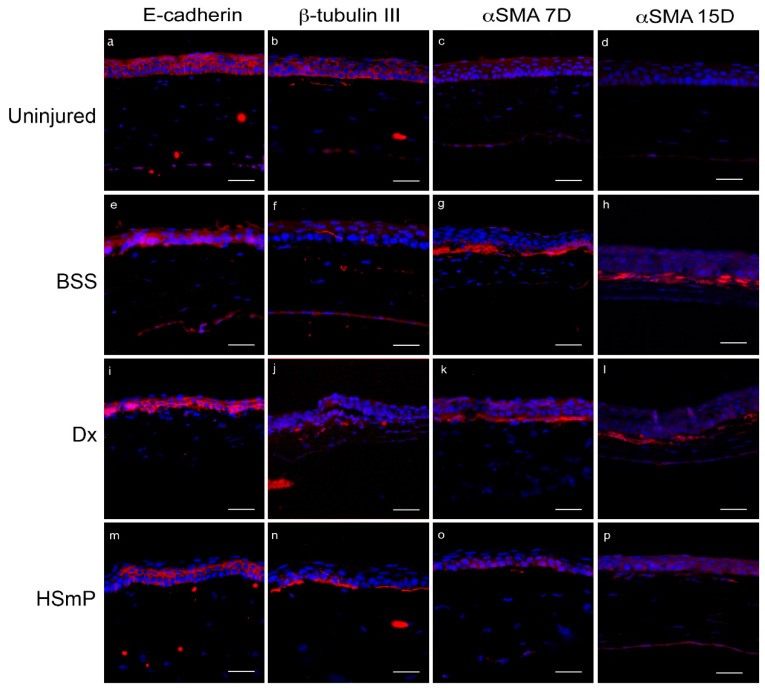
Expression of epithelial, myofibroblast and innervation markers. Column 1: Epithelium cytoarchitecture 7 days after injury. Uninjured corneas showed a structured epithelium with E-cadherin staining restricted to the periphery of the cells, forming a typical network pattern (**a**). The BSS group (**e**) presented irregularities in the epithelium and diffuse E-cadherin staining. Corneas treated with Dx exhibited E-cadherin staining at the superficial layers of the epithelium but it was absent from basal layers (**i**). HSmP-treated corneas (**m**) presented a correctly stratified epithelium with normal E-cadherin staining. Column 2: Intraepithelial nerve regeneration 7 days after injury in cross-section. The subbasal plexus is easily recognizable in uninjured corneas at the bottom of basal epithelial cells (**b**). Numerous nerve terminals rose to the epithelial surface. The general morphologies of subbasal plexus and intraepithelial terminals were similar between uninjured and HSmP groups (**n**). BSS- (**f**) and Dx-treated (**j**) corneas exhibited poor density in intraepithelial nerves. Column 3: Myofibroblast transformation 7 days after injury. Uninjured corneas had negative immunolabeling for αSMA (**c**). In contrast, many αSMA+ myofibroblasts could be observed in BSS and Dx groups (**g**,**k**), while HSmP (**o**) presented a low number of αSMA+ cells. Column 4: Myofibroblast transformation 15 days after injury. Uninjured corneas did not contain any αSMA+ myofibroblasts (**d**). HSmP-treated corneas exhibited a reduced number of myofibroblasts (**p**) compared to BSS (**h**) and Dx (**l**). Nuclei were counterstained with DAPI (scale bars: 50 µm).

Two days after laser ablation, the density of apoptotic TUNEL^+^ cells was markedly reduced in all groups (Table 2). Although Dx- and HSmP-treated corneas showed a high number of apoptotic cells, differences from BSS were not statistically significant. The density of apoptotic cells progressively decreased to day 3, when the number of death-committed cells in the stroma decreased notably, especially in HSmP and BSS groups compared to Dx (Figure 3d,f,h). At day 7, all injured groups showed similar densities to uninjured animals (Table 2) and dying cells were found exclusively at the outermost layer of the epithelium, resembling the normal superficial cell turnover process in the cornea (see Figure 4a,b).

#### 3.3.3. Epithelial Morphology

Figure 4a shows the normal cytoarchitecture of mouse corneal epithelium consisting of four to five layers of cells. Using E-cadherin immunofluorescence to visualize the microscopic structure of the epithelium, corneas treated with HSmP showed morphology similar to that for uninjured corneas 7 days after injury (Figure 4m). The inner border of the epithelium seemed to be regular except for some punctual alterations. In contrast, corneas treated with BSS or Dx showed an irregular cytoarchitecture in the epithelium (Figure 4e,i). Epithelial layers were undefined. At day 7, only the HSmP group had a normal layered epithelium, while the rest of the groups showed supernumerary epithelium layers and the shape of the cells and nuclei was spherical. E-cadherin was not present at the basal border of the epithelium in Dx- or BSS-treated corneas.

#### 3.3.4. Myofibroblast Formation

There were noticeable differences regarding fibrotic transformation and the average number of contractile cells in the cornea depending on the treatment received. There were significantly less αSMA^+^ myofibroblasts in HSmP-treated corneas at day 7 (Figure 4o) compared to the BSS (*p* < 0.001; Figure 4g) and Dx groups (*p* < 0.05; Figure 4k) (see Table 1). Interestingly, only HSmP-treated corneas kept a reduced number of myofibroblasts at day 15, while Dx and BSS presented a thick layer of αSMA-positive cells in the anterior stroma (Figure 4h,l,p).

#### 3.3.5. Nerve Regeneration

HSmP-treated corneas exhibited a significantly higher number of intraepithelial nerve terminals in cross-section compared to Dx (*p* < 0.05) and BSS (*p* < 0.01) groups (Figure 4f,j,n). At day 7, HSmP-treated corneas showed approximately one half of the intraepithelial nerve density found in uninjured mice (60.5 ± 8 terminals/mm in HSmP vs. 110.6 ± 4.9 terminals/mm in uninjured corneas) (see Table 1 for details).

**Figure 5:**
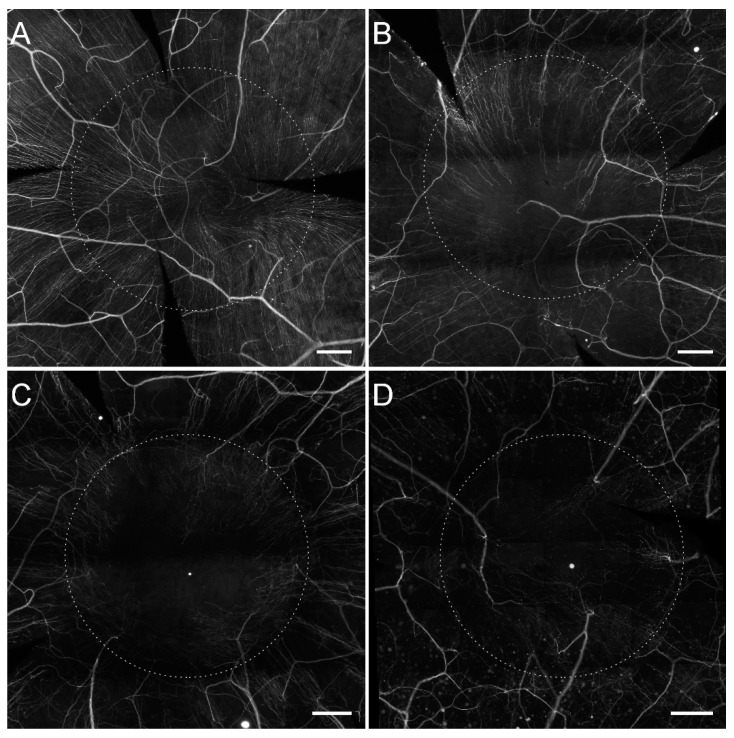
Nerve regeneration in whole-mount preparations of the cornea. (**A**) Example of an uninjured cornea showing intact β-tubulin III^+^ corneal sensory innervation with a dense subbasal plexus. (**B**) HSmP-treated corneas showed higher numbers of regenerating axons in the ablated zone (dashed line) than Dx (**C**) and BSS (**D**) groups. Furthermore, regenerating fibers in the HSmP group reached a greater distance from the edge of the lesion (scale bars: 200 µm).

**Figure 6:**
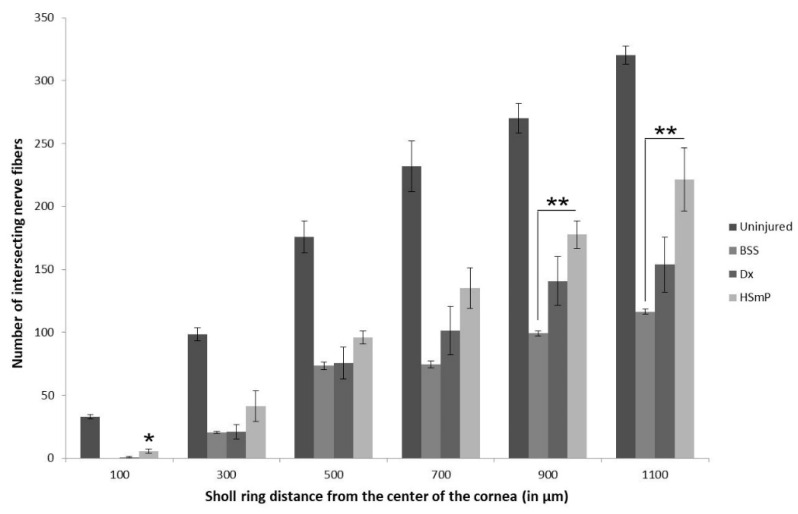
The graph shows the number of fibers intersecting Sholl’s rings at each distance point. (* = *p* < 0.05; ** = *p* < 0.01). Differences for uninjured corneas compared to the rest of the groups were omitted from the graph. They were *p* < 0.001 with Dx and BSS at all distances. Differences between the uninjured group and HSmP group were *p* < 0.001 at 100 and 500 µm and *p* < 0.01 at 300, 700, 900 and 1100 µm.

Sholl analysis of whole-mount preparations showed that laser ablation of the corneal surface produced a circular lesion that eliminated all sensory fibers of the subbasal plexus in the area. In addition, the density of subbasal fibers at the periphery of the lesion was significantly reduced 7 days after ablation. While an uninjured cornea showed an average of 320.40 ± 7.12 fibers at 1100 µm, far from the center of the cornea, control animals (treated only with BSS) showed only 116.50 ± 1.96 fibers (*p* < 0.001). Nerve regeneration in the injured area was observed at 7 D in all groups (Figure 5). Regenerating fibers sprouted from nerve stumps at the edge of the lesion located 700 µm from the center of the cornea and progressed radially toward the center. HSmP-treated eyes showed a significantly higher number of nerve fibers in the center of the cornea at day 7 than Dx and BSS groups (*p* < 0.05). Furthermore, HSmP-treated corneas showed more fibers in the periphery of the lesion than the Dx and BSS groups (*p* < 0.01). There was no statistical difference between the Dx and BSS groups (Figure 6).

## 4. Discussion

The results obtained in this study suggest beneficial effects from the application of HSmP on corneal lesions induced by a laser surgical ablation. The surgical technique used in this study produced uniform and reproducible lesions, reducing the variability in the evaluation of the wound closure defect [17,30,50,51,52,53].

HSmP is a poly(carboxylmethylglucose sulfate) that is supplied as the RGTA Cacicol^®^—a sterile, ready-to-use, single-dose solution—and the treatment requires only a short dosage routine (2–3 days each) [1]. Topical application of HSmP or its vehicle (Dx) on the ocular surface of mice did not affect the integrity of epithelial junctions, as supported by our results, showing no retention of fluorescein on uninjured eyes. Our results showed marked improvement in tissue repair when comparing HSmP to its vehicle and BSS.

One of the putative applications of HSmP as a heparin sulfate mimetic regenerative agent is to restore the ECM. While epithelial erosions in the cornea have a good prognosis, stromal damage is difficult to treat due to its extremely organized cytoarchitecture. Good recovery of the wound requires the restoration of the lost stromal tissue. In our model of lesion, as occurs in deep corneal traumas [3], a large amount of epithelial and stromal tissue was removed from the cornea.

Epithelial alterations induce changes in the organized stromal ECM [54], and a rapid recovery of the corneal epithelium is supposed to facilitate a good healing process [4,55]. Topical addition of one drop of HSmP immediately after laser ablation resulted in a rapid and efficient closure of the epithelial defect, significantly faster than Dx or BSS treatments (*p* < 0.01). Moreover, most of the eyes treated with HSmP (60%) were completely reepithelized as rapidly as 48 h post-surgery, more than double than in animals treated with Dx and BSS, supporting the role in cell migration stimulation of high-sulfated heparan sulfate glycosaminoglycan. We found also a rapid effect in the reduction of the wounded area in the Dx-treated eyes. This result, together with the higher number of Ki67^+^ cells counted in Dx-treated corneas and the relatively low rate of apoptosis, pointed to a possible role of the vehicle (a dextran) in accelerating cell proliferation during wound healing [56]. However, excess corneal epithelial cell proliferation contributes to erosion formation [57]. Our results also demonstrated that the cytoarchitecture of the epithelium was normal at day 7 only in HSmP-treated corneas, while Dx- and BSS-treated epithelia showed disorganization and altered numbers of layers, together with discontinuities in the basal layer and lack of E-cadherin expression, indicating contact between epithelial cells and stroma. Even with an elevated division rate, the number of completely closed lesions at 2 D in the Dx group was only 26%, similar to the BSS control group (30%).

On the other hand, HSmP did not demonstrate a significant increase in the number of dividing cells while also promoting a higher removal of supernumerary cells. Apoptosis appears to act in a coordinated manner with proliferation during regenerative processes to achieve a balance in cell number during healing [9,10,58]. In this sense, HSmP seems to facilitate the correct balance between division and cell removal, and this results in fast wound closure with a correct epithelial architecture.

Our results support the hypothesis that good epithelial healing promotes better transparency of the cornea. Contact between epithelial cells and stroma causes myofibroblast transformation induced by TGFβ secreted from the damaged epithelium [53,54,59]. In close relation with the speed and efficiency of epithelial healing, HSmP-treated eyes showed very good recovery of transparency levels from the third day of treatment. HSmP exerted a clear effect reducing the presence of myofibroblasts in the stroma and showed the lowest proportion of αSMA^+^ cells, significantly less than in Dx (*p* < 0.01) and BSS (*p* < 0.001) groups. In this sense, HSmP has been previously reported to have an antifibrotic effect by decreasing collagen III synthesis and improving collagen reorganization. HSmP also inhibits proteolytic enzymes in vitro [60]. In addition, the relatively reduced number of proliferative cells found in mice treated with HSmP may contribute to reducing the amount of myofibroblasts in a similar way to that using mitomycin C, which successfully reduces scarring after PRK due to its antineoplastic properties [57]. On the other hand, both Dx and BSS treatments showed poor transparency values at the end of the experiment (15 D). Dx-treated eyes showed an initial reduction of the opacity at 7 D, possibly due to a high rate of apoptosis between 3 D and 7 D (Table 2). However, while the number of apoptotic cells reached a normal value (7 D), the number of αSMA^+^ cells was significantly higher than in the HSmP group.

The presence of the myofibroblast phenotype in the tissue is also related to a reduced deposition of matrix [54], and we noticed a possible relation between the number of myofibroblasts in the cornea and the recovery of the stromal thickness. HSmP-treated corneas showed the lowest number of αSMA myofibroblasts and also exhibited similar thickness as uninjured corneas. On the other hand, corneas treated with Dx and BSS presented a corneal thickness of about one half of the uninjured thickness.

These results point to an effect of HSmP drops on the stabilization and promotion of ECM deposition and tissue remodeling in the cornea. HSmP has been shown to mimic the action of heparan sulfate molecules, thereby recreating a matrix microenvironment in which cells can migrate and multiply [1].

Interestingly, this study also showed promising results for HSmP for nerve regeneration, which is crucial to the survival of the corneal tissue [61,62,63]. We found a clear neuroregenerative effect of HSmP, with a marked significant difference from animals treated only with Dx (*p* < 0.01) and BSS (*p* < 0.001), which failed to promote rapid axonal regrowth. The density of intraepithelial nerve fibers in HSmP-treated corneas reached more than 54% of that measured in uninjured eyes as rapidly as 7 days after injury. The normal process of nerve regeneration in the cornea (for instance, the BSS group) only receives about 30% of the normal density of nerves in the epithelium. We noticed that the vehicle (Dx) by itself did not promote nerve regeneration and it was statistically equivalent to BSS. Aifa et al. (2012) described the efficacy of RGTA (HSmP) drops in healing chronic corneal lesions in humans. In a rabbit experimental model of alkali-induced corneal lesions, administration of RGTA ophthalmic solution was found to enhance the speed and quality of healing, restoring almost-normal corneal histology after only 1 week [35]. The use of HSmP has also been reported as treatment in corneal neurotrophic ulcers with good results [1], and its application after a laser-induced surgery as nerve regenerating agent could probably avoid the undesirable effects of the addition of growth factors, such as pain sensitization, induction of angiogenic episodes or excessive proliferation of cells [4,64,65].

Both the enhanced epithelial closure and rapid reinnervation processes observed after HSmP treatment in comparison with Dx and BSS could aid in avoiding painful sensations in patients after corneal injuries. Supporting this hypothesis, it has been shown that HSmP can also alleviate pain and symptoms in patients after PRK and, thus, it could be a useful intervention after PRK surgery [38]. In addition, HSmP showed a unique matrix regeneration effect not found with other treatments, restoring the stromal thickness to values present previous to the injury.

## 5. Conclusions

Taken altogether, the results of this study suggest that HSmP topically applied onto the ocular surface presents important and significant advantages over other treatments used in clinical practice, such as autologous serum or immunosuppressant drugs, modulating wound healing with regeneration of the stromal tissue, participating in the correct assembly of epithelial cells, promoting nerve regeneration and efficiently avoiding myofibroblast formation and opacity. Treatment with HSmP has been shown to heal the epithelial defect significantly faster, as well as inhibit myofibrobalst scarring. Our results support improved reinnervation of the injured area in the early stages of wound healing. These effects of HSmP on nerve regeneration and wound healing suggest that heparan sulfate mimetic polymers could help restore the integrity of the ocular surface after injury in a very effective manner. However, due to variability in nerve detection by immunolabeling of corneal transversal sections to the complexity of the corneal innervation, the results presented here should be considered with caution. A more in-depth analysis of each of the items in this study should be performed to better understand the role of synthetic heparan sulfate mimetic polymers in corneal wound healing.

## Figures and Tables

**Table 1:** Measurements of histomorphometric parameters at 7 D.

Group	Corneal Thickness (in µm ± SEM)	Myofibroblasts (Number of α-SMA^+^ Cells ± SEM)	Intraepithelial Nerve Density (β-Tubulin III^+^ Fibers/mm ± SEM)
Uninjured	105.47 ± 1.60	0	110.61 ± 4.89
BSS	51.95 ± 2.59	11.8 ± 1.49	33.26 ± 3.78
Dx	61.16 ± 5.23	7.92 ± 0.45	37.32 ± 2.83
HSmP	98.94 ± 1.62	4.92 ± 0.60	60.49 ± 8.05

**Table 2:** Values of cell density attending to proliferation and apoptosis during the corneal wound healing.

Group	Proliferating Cells (Ki67^+^ Cells/mm ± SEM)				Apoptotic Cells (TUNEL^+^ Cells/mm ± SEM)			
	D1	D2	D3	D7	D1	D2	D3	D7
Uninjured	-	-	-	48.06 ± 4.09	-	-	-	18.01 ± 0.72
BSS	272.29 ± 8.13	77.42 ± 2.72	65.30 ± 5.34	47.36 ± 1.73	256.03 ± 12.49	42.35 ± 2.62	31.60 ± 4.54	15.90 ± 0.75
Dx	273.16 ± 6.96	55.11 ± 1.73	77.52 ± 2.97	58.07 ± 1.84	169.51 ± 16.82	62.67 ± 3.00	63.99 ± 26.86	18.44 ± 2.09
HSmP	204.57 ± 17.89	44.70 ± 2.32	44.15 ± 6.24	51.65 ± 4.14	143.97 ± 25.57	61.15 ± 25.93	31.53 ± 8.73	16.50 ± 2.50

## Data Availability

The data presented in this study are contained within the article.
